# A Single Subcutaneous Dose of BMD-1141 Stimulates Hair Regrowth Comparable to Daily, High-Dose Ruxolitinib in a Mouse Model of Alopecia Areata

**DOI:** 10.3390/cells14161251

**Published:** 2025-08-14

**Authors:** Robert Gensure, Brett King, Stephen Sikkink, Andrei Mardaryev, Robyn Goforth

**Affiliations:** 1 Dartmouth Health Children’s, Lebanon, NH 03766, USA; 2 Independent Researcher, Fairfield, CT 06430, USA; brett.aking@gmail.com; 3 School of Chemistry and Biosciences, University of Bradford, Manchester BD7 1DP, UK; s.k.sikkink@bradford.ac.uk; 4 Revenue Jersey, St. Helier, Jersey JE4 8PF, UK; a.mardaryev@gov.je; 5 BiologicsMD, New Haven, CT 06511, USA; robyn.goforth@biologicsmd.com

**Keywords:** alopecia areata, hair cycle stimulator, JAK inhibitor, BMD-1141

## Abstract

Alopecia areata (AA) is an autoimmune disorder of hair loss resulting from a T-cell mediated attack on hair follicles. Three Janus kinase (JAK) inhibitors have been approved for the treatment of moderate-to-severe alopecia areata; however, safety concerns for immunosuppressive therapy have limited their use. We previously demonstrated that BMD-1141, consisting of parathyroid hormone (PTH) fused to a collagen-binding domain (CBD) (PTH-CBD) improved hair retention, increased anagen hair follicles counts, and reduced hair follicle dystrophy in C3H/HeJ-engrafted mice). We now compare the effects of a single subcutaneous injection of BMD-1141 with the daily, high-dose, oral administration of the JAK inhibitor ruxolitinib on anagen hair follicle counts and hair regrowth in C3H/HeJ-engrafted mice. BMD-1141-treated mice exhibited a significant increase in anagen hair follicle counts (*p* < 0.05) and enhanced hair regrowth compared to ruxolitinib-treated mice after 8 weeks. Hair follicles from the BMD-1141-treated mice showed increased beta-catenin, consistent with a mechanism of stimulating the anagen transition of hair follicles, and did not increase immune cell infiltration. Thus, a single subcutaneous dose of BMD-1141 stimulated hair regrowth comparable to daily ruxolitinib, apparently by stimulating the hair cycle, rather than inhibiting the autoimmune response.

## 1. Introduction

Alopecia areata (AA) is a chronic form of non-cicatricial hair loss resulting from an autoimmune disorder that impacts hair follicles. It results in either patchy or complete hair loss on the scalp. More severe forms result in alopecia universalis, with the loss of all body hair. AA is a common cause of hair loss, affecting around 2% of the population, or around 6.6 million people in the US [1]. It affects males and females equally and occurs in both children and adults. Hair loss from AA has significant social implications which result in emotional distress. Individuals with AA are often misidentified as having a more serious illness, such as cancer. In addition, while male-pattern or androgenetic hair loss is generally socially accepted, hair loss caused by AA, particularly in females, can be especially distressing. The emotional toll can be profound, with some patients experiencing such severe dysphoria that it increases the risk of suicide ideation [2,3,4].

Mechanistically, AA is an autoimmune disease primarily characterized by chronic inflammation at the hair follicle. However, its underlying pathophysiology is complex. It is commonly suggested that changes in T-helper 1/interferon function are a primary driver of AA, but more recent studies suggest the involvement of additional immune mediators [5,6,7]. Under normal conditions, hair follicles possess immune privilege, protecting them from immune responses. However, this privilege is lost in AA. Although anagen-phase hair follicles are the primary targets, there is some evidence that hair follicles in other phases of the hair cycle may also be affected by the immune response [8]. Regardless of the stage in the hair growth cycle where the immune attack takes place, the damaged hair follicles become dystrophic, and the hair shaft is lost [9], resulting in visible hair loss. Prior to 2022, there were no approved therapies, but treatment with glucocorticoids, methotrexate, and topical sensitizers were common therapeutic approaches with limited efficacy [10]. Current management is typically based on the extent of hair loss. Topical treatments and intralesional steroid injections are still typically used as first-line therapies for mild cases, whereas broad immunosuppressants, such as the newly approved JAK inhibitors, are more likely to be used in the treatment of more severe cases. However, whether evaluating therapies that center on glucocorticoid administration, either topically or injected at the sites of hair loss, or the use of the JAK inhibitors to reduce the global immune reaction, response rates are typically around 30–45% [11]. Due to the limited efficacy of current therapies, many patients rely on careful hair styling to conceal patches of hair loss or wear wigs to cover more significant hair loss or baldness.

A key reason for the relatively low response rate, as evaluated by hair regrowth, is that while JAK inhibitors are effective at reducing the immune response in alopecia areata, they do not promote the repair or regeneration of hair follicles. As such, JAK inhibitors rely on the body’s own renewal mechanisms for the regrowth of hair. This leads to long delays in the maximal treatment response; often the maximum response rate is calculated after 8–12 months of treatment [11]. The long timelines until response, coupled with nonresponders making up 50–70% of the study population in large clinical trials [11], means there is still a significant portion of individuals with alopecia areata that lack effective therapies. In addition, the US Food and Drug Administration has placed a black box warning on the JAK inhibitor class due to safety concerns, which include secondary malignancies, infections, the risk of thrombosis, and drug interactions, based on data from studies on rheumatoid arthritis and other dermatological conditions [12]. Therefore, the long-term safety of JAK inhibitors will need to be further examined in the treatment of alopecia areata [13].

While the approved JAK inhibitors and most therapies in development for the treatment of alopecia areata focus on suppressing the autoimmune attack, an alternative approach is the stimulation of the hair cycle. Hair cycle stimulators could potentially have several key advantages over immunosuppressive therapies, including the promotion of the more-rapid and complete regrowth of hair, as well as potentially avoiding the safety consequences of global immune suppression. To date, the only reliable hair cycle stimulator is cyclosporin, which has shown efficacy in promoting the regrowth of hair [14]. However, cyclosporin is also a broad-spectrum immune suppressant with a similar side-effect profile to JAK inhibitors, leading to an unfavorable benefit–risk profile for routine use. On the other hand, parathyroid-hormone-related protein (PTHrP) is also a hair cycle stimulant. It acts by increasing beta-catenin in the stem cells found in the bulge region of the hair follicles, stimulating the anagen transition [15]. However, most PTHrP analogs share a functional similarity to PTH and result in increased serum calcium levels, leading to the potential for hypercalcemia. BMD-1141 is a skin-targeted PTHrP analog, attached to a collagen-binding domain, which has an intrinsic collagen binding capacity that concentrates and retains the analog in the skin. Here we demonstrate that a single dose of BMD-1141 promotes hair regrowth in the C3H/HeJ-engrafted mouse model of alopecia areata, with effects comparable to high-dose, daily ruxolitinib, a JAK inhibitor.

## 2. Materials and Methods

Toxicology Studies: All the studies were completed under IACUC approval. In Phase I (Dose Escalation), BMD-1141 was administered to sixteen Sprague-Dawley rats (8 males and 8 females) via subcutaneous injection at dose levels of 0.32, 0.96, 1.92, and 6 mg/kg (Appendix A, Table A1). A stepwise dosing approach was employed, allowing 3 to 4 days between successive doses to ensure tolerability. Each animal received a dose volume of 5 mL/kg and was observed for 7 days post-administration. On Day 8, the animals were euthanized for blood sample collection and necropsy. In Phase II (Dose Confirmation), twenty-four rats (12 males and 12 females) were dosed with BMD-1141 at 1, 3, and 10 mg/kg on Days 1 and 9, with a control group receiving no treatment (Appendix A, Table A3). After the final dose, the animals were observed for 24 h before undergoing blood sample collection and necropsy on Day 10.

The rats were housed in groups of up to three of the same sex (except during designated procedures) in transparent plastic bins equipped with automatic watering systems. The animal room environment was controlled (target temperature: 21 ± 3 °C; relative humidity: 50 ± 20%; with 12 h light/dark cycles and a minimum of 10 air changes per hour). Temperature and humidity were continuously monitored. The animals were provided with a standard certified rodent chow (Envigo Global 18% Protein Rodent Diet #2018C, Inotiv, West Lafayette, IN, USA) and purified municipal tap water ad libitum, except during designated procedures. Non-dietary items and certified treats were offered as environmental enrichment, and veterinary care was available throughout the study as needed.

At the onset of treatment in Phase I, the rats’ body weights were slightly above the target range (250 to 325 g for males and 175 to 250 g for females), but this deviation was considered to have no impact on the study results. The animals were randomized into dose groups by block randomization based on body weight, with males and females randomized separately. Hematology and clinical chemistry assessments were performed at the study’s termination on Day 8 (Phase I) and Day 10 (Phase II), with blood samples collected from the abdominal aorta. The animals were fasted overnight before blood sample collection. The necropsy included both external and detailed internal examinations.

Efficacy Studies: All the studies were completed under IACUC approval. Hair loss consistent with alopecia areata (AA) occurs naturally in approximately 20% of aged female C3H/HeJ mice, with an increased prevalence observed in ex-breeding females obtained from The Jackson Laboratory, Bar Harbor, ME, USA [16]. AA can be reproducibly induced in a larger proportion of C3H/HeJ mice through full-thickness skin graft transplantation from affected donors to 10–16-week-old female recipients [16].

To generate sufficient donor mice for the study, 100 ex-breeder C3H/HeJ females (aged approximately 7–9 months) were purchased from The Jackson Laboratory and housed in the Bradford University vivarium (Bradford, West Yorkshire, UK) until they developed AA to a degree suitable for transplantation, following previously established protocols by Sandberg et al. [reference needed]. Donor mice exhibiting active AA lesions were euthanized, and full-thickness dorsal skin grafts (approximately 1–2 cm^2^) were excised under sterile conditions. The grafts were trimmed to remove excess subcutaneous fat and immediately placed in sterile saline to maintain viability. Recipient C3H/HeJ mice were anesthetized, and a corresponding section of dorsal skin was surgically excised to create a graft bed. The donor skin was then positioned onto the recipient site and secured using surgical sutures. Postoperative care included analgesic administration and routine monitoring for signs of infection or graft rejection.

The recipient mice (*n* = 80) were housed for 31 weeks in a controlled environment (temperature: 21 ± 3 °C; relative humidity: 50 ± 20%; 12 h light/dark cycle), with the continuous monitoring of environmental conditions. The animals had ad libitum access to a low-isoflavone rodent diet and purified water, except during experimental procedures. Veterinary care was available as needed throughout the study.

Following transplantation, the development of AA was visually assessed. Of the 80 recipient mice, 44 developed moderate-to-severe AA. These mice were stratified into seven treatment groups (*n* = 6 per group) using a randomized allocation strategy to ensure the balanced distribution of the hair loss severity across groups. The hair loss severity varied within each group, ranging from patchy to total alopecia. The treatment groups were as follows: control (no treatment, Group 1); BMD-1141 administered subcutaneously as a single dose at 40, 160, 320, 640, or 1000 µg/kg (Groups 2–6); and ruxolitinib administered via oral gavage daily at 50 mg/kg in 0.5% methylcellulose (Group 7). Two mice from the ruxolitinib group were removed within the first two weeks due to health complications.

The mice were photographed under isoflurane anesthesia at baseline (Day 0), at the midpoint (Week 4), and at the end of treatment (Week 8). Hair loss was quantified using a semi-automated image analysis in FIJI (ImageJ 1.53) software. Briefly, dorsal images from Days 0, 28, and 56 were standardized by cropping to equal dimensions and converting to 8-bit grayscale. Haired regions were identified using a threshold analysis, and the hair-covered area was measured in cm^2^. The data were compiled and analyzed using Prism software 9.4.0, with the standard deviations (SDs) represented in error bars on the graphical outputs.

For the histological analysis, dorsal skin from AA-affected mice was fixed in 10% neutral-buffered formalin (NBF), paraffin-embedded, and sectioned at 7 µm thickness. Hematoxylin and eosin (H&E)-stained sections were scanned using a digital slide scanner. Three representative full-thickness skin regions (head, mid-body, and tail) were selected for follicle scoring. Two independent observers classified follicles as dystrophic anagen, normal anagen, or unknown (if the orientation or classification was uncertain). Inflammatory cell infiltrates were scored based on their presence in cropped images: mild (+/1 image), moderate (++/2 images), or severe (+++/3 images). The scores were summed across regions to generate a whole-mouse inflammatory profile. The combined total follicle count (dystrophic + normal + unknown) was also recorded. Mean values were derived from observer counts, and statistical analyses were performed using Prism software.

For immunohistochemistry (IHC), dorsal skin samples were snap-frozen in liquid nitrogen and embedded in an OCT medium. Sections (10 µm) were cut, dried at 4 °C overnight, and fixed in acetone (β-catenin) or a 1:1 acetone/methanol mixture (CD4/CD8a) at −20 °C for 10 min. After air drying, the sections were stored at −80 °C until staining. Before staining, the slides were rehydrated in PBS and blocked in 10% donkey serum for 1 h. Primary antibody incubation was performed overnight at 4 °C in 1% donkey serum. Antibodies included rabbit anti-mouse β-catenin [E247] (Abcam, Cambridge, UK, Cat# ab32572, 1:250 dilution), rat anti-mouse CD4 [Clone: GK1.5] (Thermo Fisher, Waltham, MA, USA, Cat# 15286747, 1:10 dilution), and rat anti-mouse CD8a [Clone: 53–6.7] (Thermo Fisher, Waltham, MA, USA Cat# 15236777, 1:10 dilution). The sections were rinsed three times for 10 min in PBS before incubation for 1 h with secondary antibodies diluted 1:100 in 1% donkey serum. Secondary antibodies included Alexa Fluor™ 488 donkey anti-rat (for CD4 and CD8a) and Alexa Fluor™ 488 donkey anti-rabbit (for β-catenin). Following three final PBS washes (10 min each), the slides were mounted in a DAPI-containing mounting medium and cover-slipped.

Fluorescent imaging was conducted using a Leica (Wetzlar, Germany) DM2500 LED microscope with UV and FITC filters. Imaging magnifications were ×10 (β-catenin) and ×20 (CD4 and CD8a), maintaining consistent exposure settings across slides. Where possible, two follicular regions per sample were imaged, with higher magnification (×40) used to capture regions of interest. The images were processed using FIJI software.

## 3. Results

**Figure 1:**
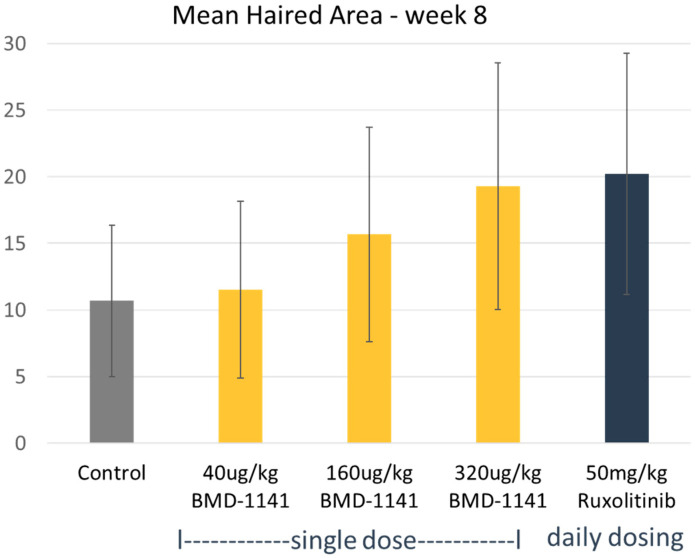
Comparative Efficacy. C3H/HeJ mice were engrafted with full-thickness skin from mice with alopecia areata. After the development of alopecia, the mice were treated with either a single subcutaneous dose of BMD-1141, daily oral ruxolinitib, or a vehicle control, as indicated. The mean hair area was assessed after 8 weeks of treatment.

**Figure 2:**
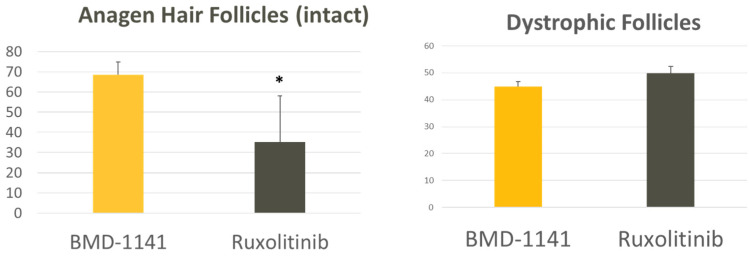
Hair Follicle Counts. C3H/HeJ mice were engrafted with full-thickness skin from mice with alopecia areata. After the development of alopecia, the mice were treated with either a single subcutaneous dose of BMD-1141 (at various doses) or daily oral ruxolinitib. After 8 weeks, the animals were sacrificed, and dorsal skin was harvested and sectioned at 7 µm thickness. Hematoxylin and eosin (H&E)-stained sections were prepared, and follicles were classified as dystrophic anagen, normal anagen, or unknown. The average hair follicle counts are compared between a pool of animals treated with any dose of BMD-1141 vs. ruxolitinib (* *p* < 0.05 by Student’s *t*-test).

**Figure 3:**
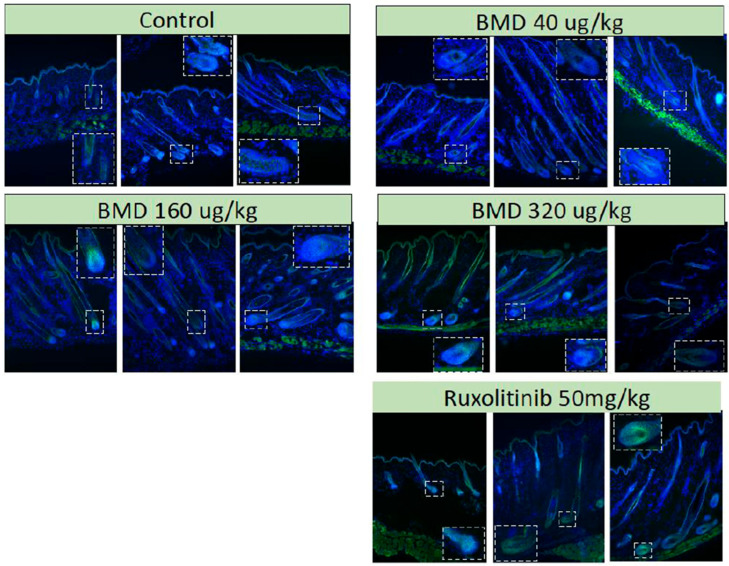
Beta-Catenin. C3H/HeJ mice were engrafted with full-thickness skin from mice with alopecia areata. After the development of alopecia, the mice were treated with either a single subcutaneous dose of BMD-1141 (at various doses) or daily oral ruxolinitib. After 8 weeks, the animals were sacrificed, and dorsal skin was harvested and sectioned at 7 µm thickness. The dorsal skin samples were sectioned (10 µm) and were incubated with rabbit anti-mouse β-catenin. Fluorescent imaging was conducted at ×10 magnification. The immunofluorescence for β-catenin is shown in green.

Treatment with BMD-1141 led to observable, dose-dependent increases in hair growth, as evaluated by the mean hair area, showing similar results to the ruxolitinib-treated group, at the expected therapeutic dose of 320 mcg/kg (Figure 1), though the overall changes did not reach statistical significance, even in the high-dose ruxolitinib group; this is likely due to the substantial initial hair loss at the start of the study. However, as a hair cycle stimulator, BMD-1141 was expected to produce a greater increase in the hair follicle number compared to ruxolitinib. Consistent with this, the number of anagen hair follicles was higher in the pooled BMD-1141-treated groups than in the ruxolitinib group, with the greatest differences seen at doses ≥32—mcg/kg, after 8 weeks of treatment (Figure 2, *p* < 0.05). Additionally, there was a trend towards fewer dystrophic hair follicles following the BMD-1141 treatment compared to ruxolitinib, consistent with the recovery of damaged hair follicles (Figure 2). Supportive of the proposed mechanism of action, immunohistochemistry revealed that there were evident, dose-dependent increases in beta catenin after the treatment with BMD-1141 as compared to those found in the control and the ruxolitinib-treated animals (Figure 3).

**Figure 4:**
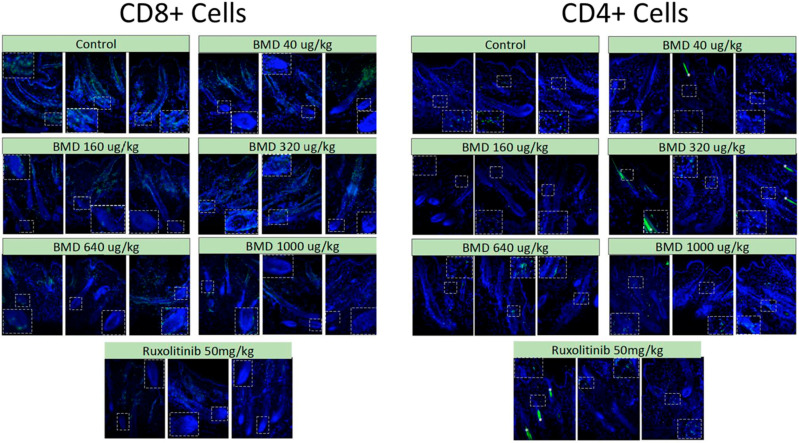
Immune Response. C3H/HeJ mice were engrafted with full-thickness skin from mice with alopecia areata. After the development of alopecia, the mice were treated with either a single subcutaneous dose of BMD-1141 (at various doses) or daily oral ruxolinitib. After 8 weeks, the animals were sacrificed, and dorsal skin was harvested and sectioned at 7 µm thickness. The dorsal skin samples were sectioned (10 µm) and were incubated with rat anti-mouse CD4 or rat anti-mouse CD8a. Fluorescent imaging was conducted at ×20 magnification. The immunofluorescence for CD4 or CD8a is shown in green.

While there were potential concerns that the increase in the number of anagen hair follicles following BMD-1141 treatment might provide more substrates for immune attack and exacerbate the immune response, immunohistochemistry showed no evidence of this. There were no apparent differences in the immune response between the BMD-1141 and ruxolitinib groups, even after 8 weeks of therapy (Figure 4). This suggests that the anagen hair follicles may not be the trigger for the immune response, but rather the target for damage from an ongoing autoimmune process.

The thorough evaluation of the toxicology profile of BMD-1141 is essential when comparing its efficacy to ruxolitinib and the JAK inhibitor class. Ruxolitinib is a potent immunosuppressive agent; thus, while effective in treating hair loss for some patients, it also exposes patients to significant systemic effects, such as severe infections, malignancies, and other safety risks. Understanding the safety and tolerability of BMD-1141, including its potential to avoid any immune-related complications, is critical to assessing its overall therapeutic value. In the single-dose subcutaneous administration toxicology testing of BMD-1141 in rats, doses of up to 6 mg/kg were well tolerated. No clinical signs of toxicity, changes in body weight, or alterations in food consumption were observed during the 7-day observation period. Additionally, there were no significant findings in clinical pathology, organ weight measurements, or macroscopic and microscopic evaluations that could be attributed to BMD-1141 (Appendix A, Table A2). The repeat-dose administration of BMD-141 at doses of 1 to 10 mg/kg on Days 1 and 9 also resulted in no observable clinical signs of toxicity, and no significant changes in body weight, food consumption, or hematology were noted. A mild dose-dependent increase in calcium levels was observed in both male and female rats with repeat dosing at the 3 and 10 mg/kg doses, which was not clinically significant (Appendix A, Table A2). Microscopic findings included minimal-to-moderate renal lesions (tubular basophilia, tubular dilation, pelvic dilation, and corticomedullary mineralization) at the highest dose, and mild diffuse subcutaneous hemorrhage (bruising) at the final injection site. These findings were not considered to be clinically significant due to their low severity and incidence. Based on these results, BMD-1141 was well tolerated, with a maximum tolerated dose (MTD) of 10 mg/kg per every 9 days, the highest feasible dose given the current formulation. Given that the therapeutic dose is the 320 mcg/kg to 1 mg/kg once every one to three months, the initial toxicology profile is robustly positioned.

## 4. Discussion

In an animal model of alopecia areata (C3J-HeJ engrafted mice), BMD-1141 promoted notable hair regrowth, similar to the JAK inhibitor ruxolitinib, in a dose-dependent manner after 4 and 8 weeks of treatment, despite many of the animals having nearly complete alopecia prior to therapy. BMD-1141 also showed a significant (*p* < 0.05) increase in the number of anagen hair follicles compared to ruxolitinib after 8 weeks of treatment and showed a trend towards fewer dystrophic hair follicles. There was no increase in the immune reaction observed after 8 weeks of therapy with BMD-1141 as compared to ruxolitinib. Importantly, the 8-week course of treatment for BMD-1141 consisted of a single subcutaneous injection, while ruxolitinib required daily oral dosing. The only deaths in the study were in the ruxolitinib-treated group, early in the study, due to animal illness.

BMD-1141 acts as a hair cycle stimulator, working through beta-catenin stimulation in cells located in type I collagen-rich tissues that express a PTH/PTHrP receptor, a different mechanism of action than the current immune suppressant treatments for alopecia areata. BMD-1141 promotes the transition of hair follicles to the anagen phase and appears to reverse damage in dystrophic hair follicles, restoring them to normal hair follicle cycling. These effects on the hair follicle result in visible regrowth of hair, even in the presence of an ongoing autoimmune response. Importantly, the increase in hair follicles seen after BMD-1141 treatment did not augment the autoimmune response, based on a histological analysis 8 weeks after treatment. Thus, hair regrowth was achieved without systemic immunosuppression, as caused by other therapies (such as JAK inhibitors and cyclosporine) and without the reactive stimulation of the autoimmune response against the restored follicles.

Further, BMD-1141 has a long tissue residence in the skin, resulting in a favorable dosing schedule of subcutaneous injection every 1–3 months. In contrast, orally administered JAK inhibitors have a shorter half-life and must be taken daily to be effective. While BMD-1141 has a long tissue residence in skin, its serum half-life is much shorter, around 6–8 h, which reduces the risk of systemic side-effects [17]. BMD-1141 has demonstrated a favorable safety profile in animal studies, with the expected therapeutic dose being significantly lower—by an order of magnitude—than the maximum tolerated dose. Previous studies have demonstrated that BMD-1141 does not cause hypercalcemia, as observed with daily PTH treatments, except at supratherapeutic doses. Furthermore, the only significant off-target effect observed in any pre-clinical study, to date, is an increase in bone mineral density in osteoporotic animals. This is expected, but not considered to be an adverse event, as the collagen-binding activity increases the drug concentration in the bone, where it has anabolic effects similar to those seen with daily PTH treatment [18].

## 5. Conclusions

Given its unique, beta-catenin-stimulating mechanism of action, BMD-1141 holds potential for expanding the therapeutic options for individuals with alopecia areata. If designated as a first-line or second-line monotherapy, it may circumvent the need for systemic immune suppression and thereby reduce the risk of the associated serious complications. Conversely, if used in combination with other therapeutic agents, BMD-1141 could mitigate additive immunosuppression and may facilitate combination strategies that minimize the overall level of immunosuppression required clinically (the JAK-sparing effect). Given its mechanism of hair cycle stimulation and its favorable safety profile, BMD-1141 shows promise as a therapy for the treatment of other non-cicatricial alopecias, as well as alopecia areata. Future clinical studies will be conducted to elucidate the optimal dosing regimen and the long-term safety profile of BMD-1141 as a clinical therapy for the treatment of non-cicatricial alopecia.

## Data Availability

The original contributions presented in this study are included in the article. Further inquiries can be directed to the corresponding author.

## Abbreviations

The following abbreviations are used in this manuscript:

JAK	Janus Kinase
PTH	parathyroid hormone
CBD	collagen-binding domain
PTHrP	parathyroid-hormone-related protein
AA	alopecia areata

## Appendix A

### Appendix A.1. Phase I—Single Dose Escalation Study

**Table A1:** Study design.

Group	Dose (mg/kg)	Dose (mL)	Male (*n*)	Female (*n*)
1	0.32	5	2	2
2	0.96	5	2	2
3	1.92	5	2	2
4	6	5	2	2

**Table A2:** Single Dose Clinical chemistry.

Group	Dose (mg/kg)	Sex (*n*)	Day	ALT (U/L)	AST (U/L)	ALP (U/L)	Urea (mM)	Creatinine (uM)	Calcium (mM)
1	0.32	M (2)	8	33 (6.4)	111 (60.1)	116 (5.7)	5.4 (0.28)	28 (1.4)	2.51 (0.269)
		F (2)		25 (0.7)	70 (3.5)	85 (1.4)	7.4 (0.35)	36 (2.1)	2.68 (0.014)
2	0.96	M (2)	8	28 (7.1)	111 (30.4)	174 (24)	4.3 (0.00)	28 (1.4)	2.43 (0.000)
		F (2)		29 (3.5)	84 (10.6)	73 (6.4)	2.2 (0.21)	34 (6.4)	2.52 (0.007)
3	1.92	M (2)	8	38 (4.9)	141 (50.9)	108 (31.1)	6.5 (1.56)	32 (4.2)	2.54 (0.078)
		F (2)		29 (0.7)	84 (7.1)	137 (26.9)	6.5 (0.78)	33 (0.7)	2.46 (0.014)
4	6	M (2)	8	40 (9.9)	93 (17.0)	223 (15.6)	5.7 (0.42)	29 (1.4)	2.42 (0.170)
		F (2)		26 (2.8)	93 (39.6)	79 (34.6)	5.5 (0.21)	33 (4.9)	2.59 (0.035)

Anova & Dunnett.

Conclusion: “The single dose subcutaneous administration of BMD-26 at up to 6 mg/kg did not result in any clinical signs, changes in body weight or food consumption. There were no clinical pathology changes, organ weight changes, or macroscopic and microscopic findings at the end of the 7-day observation period that could be considered to be test item-related.”

### Appendix A.2. Phase II Repeat Dose (Days 1 and 9) Escalation Study

**Table A3:** Study design: repeat dose—dosed on days 1 and 9.

Group	Dose (mg/kg/day)	Dose Volume (mL) Days 1 & 9	Male (*n*)	Female (*n*)
5	0	5	3	3
6	1	5	3	3
7	3	5	3	3
8	10	5	3	3

**Table A4:** Repeat Dose Clinical chemistry.

Group	Dose (mg/kg)	Sex (*n*)	Day	ALT (U/L)	AST (U/L)	ALP (U/L)	Urea (mM)	Creatinine (uM)	Calcium (mM)
5	0	M (3)	10	32 (5.0)	105 (35.1)	195 (13.3)	5.6 (0.85)	24 (2.1)	2.55 (0.114)
		F (3)		28 (4.5)	89 (17.3	115 (26.3)	6.1 (0.56)	28 (2.3)	2.67 (0.050)
6	1	M (3)	10	27 (2.1)	121 (30.5)	171 (6.6)	6.3 (0.31)	28 (3.6)	3.03 * (0.359)
		F (3)		26 (3.5)	111 (12.2)	99 (40.4)	5.9 (1.42)	33 (7.0)	3.00 (0.132)
7	3	M(3)	10	28 (3.1)	107 (25.5)	185 (19.9)	5.4 (0.47)	25 (0.6)	3.23 * (0.144)
		F (3)		29 (10.7)	110 (20.4)	107 (49.3)	6.6 (0.81)	34 (2.5)	3.32 (0.234)
8	10	M (3)	10	32 (4.9)	105 (14.3)	193 (37.8)	5.5 (1.07)	27 (3.0)	3.91 *** (0.326)
		F (3)		23 (3.1)	90 (29.5)	122 (45.7)	5.6 (1.10)	29 (2.5)	4.07 *** (0.536)

Anova & Dunnett: * = *p* < 0.05; *** = *p* < 0.001.

Conclusion: No test-item-related changes in clinical signs, body weight, or food consumption were observed. Likewise, no hematological or organ weight changes indicative of test-item-related effects were noted at the end of the 24 h observation period following the final dose. Mild, transient increases in serum calcium were observed in the repeat dosing groups but were not considered biologically significant. Signs of local irritation at the final dosing site (e.g., dark discoloration) were observed in a subset of animals in Groups 6, 7, and 8. Minimal-to-moderate renal lesions—including tubular basophilia, tubular and pelvic dilation, and corticomedullary mineralization—were noted in Group 8 animals (10 mg/kg/occasion). Additionally, minimal-to-mild diffuse subcutaneous hemorrhage at the final dosing site was observed in Groups 7 and 8 (3 and 10 mg/kg/occasion, respectively). According to the contract research organization, these findings were not considered adverse due to their low severity and incidence.
